# Test-retest reliability of high-resolution surface electromyographic activities of facial muscles during facial expressions in healthy adults: A prospective observational study

**DOI:** 10.3389/fnhum.2023.1126336

**Published:** 2023-03-13

**Authors:** Vanessa Trentzsch, Nadiya Mueller, Martin Heinrich, Anna-Maria Kuttenreich, Orlando Guntinas-Lichius, Gerd Fabian Volk, Christoph Anders

**Affiliations:** ^1^Division Motor Research, Pathophysiology and Biomechanics, Department of Trauma, Hand and Reconstructive Surgery, Jena University Hospital, Friedrich Schiller University Jena, Jena, Germany; ^2^Department of Otorhinolaryngology, Jena University Hospital, Friedrich Schiller University Jena, Jena, Germany; ^3^Facial-Nerve-Center Jena, Jena University Hospital, Jena, Germany; ^4^Center for Rare Diseases, Jena University Hospital, Jena, Germany

**Keywords:** electromyography, reliability—reproducibility of results, face, mimic muscles, facial palsy, emotional expression

## Abstract

**Objectives:**

Surface electromyography (sEMG) is a standard method for psycho-physiological research to evaluate emotional expressions or in a clinical setting to analyze facial muscle function. High-resolution sEMG shows the best results to discriminate between different facial expressions. Nevertheless, the test-retest reliability of high-resolution facial sEMG is not analyzed in detail yet, as good reliability is a necessary prerequisite for its repeated clinical application.

**Methods:**

Thirty-six healthy adult participants (53% female, 18–67 years) were included. Electromyograms were recorded from both sides of the face using an arrangement of electrodes oriented by the underlying topography of the facial muscles (Fridlund scheme) and simultaneously by a geometric and symmetrical arrangement on the face (Kuramoto scheme). In one session, participants performed three trials of a standard set of different facial expression tasks. On one day, two sessions were performed. The two sessions were repeated two weeks later. Intraclass correlation coefficient (ICC) and coefficient of variation statistics were used to analyze the intra-session, intra-day, and between-day reliability.

**Results:**

Fridlund scheme, mean ICCs per electrode position: Intra-session: excellent (0.935–0.994), intra-day: moderate to good (0.674–0.881), between-day: poor to moderate (0.095–0.730). Mean ICC’s per facial expression: Intra-session: excellent (0.933–0.991), intra-day: good to moderate (0.674–0.903), between-day: poor to moderate (0.385–0.679). Kuramoto scheme, mean ICC’s per electrode position: Intra-session: excellent (0.957–0.970), intra-day: good (0.751–0.908), between-day: moderate (0.643–0.742). Mean ICC’s per facial expression: Intra-session: excellent (0.927–0.991), intra-day: good to excellent (0.762–0.973), between-day: poor to good (0.235–0.868). The intra-session reliability of both schemes were equal. Compared to the Fridlund scheme, the ICCs for intra-day and between-day reliability were always better for the Kuramoto scheme.

**Conclusion:**

For repeated facial sEMG measurements of facial expressions, we recommend the Kuramoto scheme.

## Introduction

Facial electromyography (EMG) is a standard tool in clinical studies and psychological experiments to assess facial muscles during specific facial expressions and to analyze the association to specific emotions (Hubert and de Jong-Meyer, 1991; Guntinas-Lichius et al., 2020; Hofling et al., 2020). The recordings in psychological settings usually are performed on the surface of facial muscles via multi-channel surface EMG (sEMG) (Tassinary et al., 1989; Barrett et al., 2019). Multi-channel sEMG is needed, because the facial muscular system forms a complex interdependent and interwoven system of facial muscles that is connected to the skin (Cattaneo and Pavesi, 2014). Hence, specific facial movements lead to a complex sEMG activation of several or even almost all facial muscles (Schumann et al., 2010, 2021; Cui et al., 2020). Actually, two different facial sEMG recording schemes are established: Most popular is the scheme developed by Fridlund and Cacioppo. They recommended to record the sEMG always from 10 specific facial and one masticatory muscle (Fridlund and Cacioppo, 1986). As an alternative, Kuramoto et al. (2019) recommended to even cover the complete face by using 21 sEMG electrodes in an EEG-like arrangement. Recently, we showed that a geometric and symmetrical sEMG recording from the entire face like it is recommended by Kuramoto et al. (2019) seems to allow a more specific distinction of facial muscle activity patterns during various facial expression tasks than the more frequently applied scheme by Fridlund and Cacioppo (Mueller et al., 2022).

In a typical psychological or clinical experiment, participants or patients are examined several times, for instance by varying the stimuli or at different days before and after an intervention. Hence, it has to be guaranteed that the respective sEMG scheme can be applied in a reliable manner to exclude a variability of the EMG recording related to variability of the electrode application. Any fixed sEMG scheme is influenced by the inter-electrode distances, crosstalk, and the influence of both on the sEMG recordings. Surprisingly, although very important, the test-retest reliability of facial high-resolution sEMG has not been analyzed in detail yet (Hess et al., 2017). If only some or single facial muscles are recorded in psycho-physiological research, the test-retest reliability can be low (Hess et al., 2017).

Therefore, we wanted to study the test-retest reliability of both the sEMG electrode schemes of Fridlund and Cacioppo and of Kuramoto et al. (2019) in the same healthy probands as in the previous study (Mueller et al., 2022). Both schemes were applied simultaneously during specific facial expressions. One sEMG session included three trials of these standardized facial expressions. The session was then repeated on the same day. The entire procedure (i.e., two sessions with three trials, respectively) was repeated 14 days later. Hence, it was possible to calculate the intra-session reliability, the intra-day reliability, and the between-day reliability of high resolution facial sEMG.

## Materials and methods

### Healthy participants

The study included the same 36 healthy adult volunteers as published recently (Mueller et al., 2022). Nineteen women and 17 men were included (age range: 18–67 years). Exclusion criteria were: neurological disease, history of facial surgery or facial trauma. The ethics committee of the Jena University Hospital approved the study (No. 2019-1539). All participants gave written informed consent to participate in the study.

### Standardization of repeated facial exercises

**FIGURE 1:**
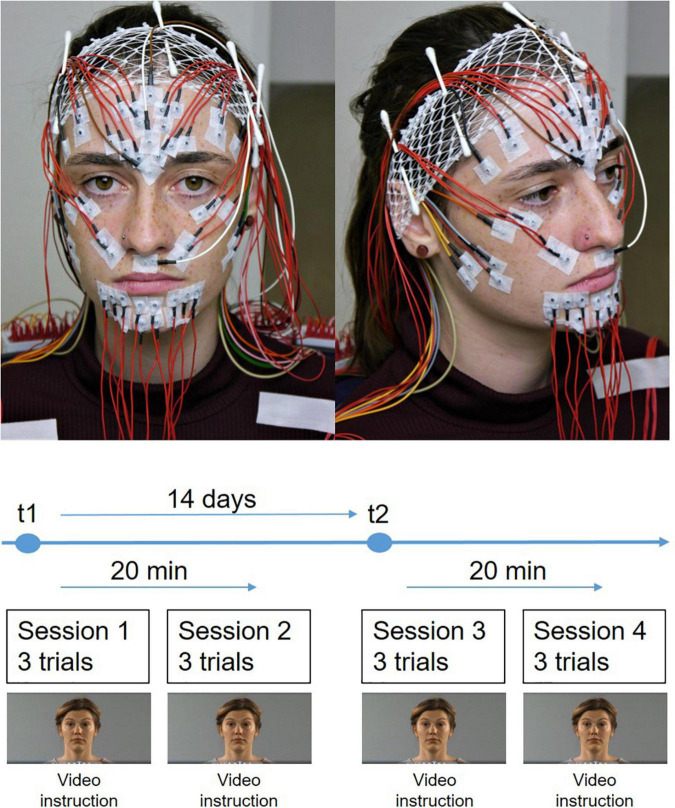
The experimental setup. **(Top)** Simultaneous application of the EMG electrode schemes after Fridlund and Cacioppo and Kuramoto on the face of the participant. **(Bottom)** Time course of the measurements. The measurements were performed at two time points (t1 and t2). The interval between t1 and t2 was 14 days. Two EMG measurement sessions were performed at each time point. The interval between both sessions was 20 min. Each sessions contained three trials for each facial movement.

The participants were instructed about the sequence of the examination. The instructions for the facial expressions presented by a video were explained. Details of the video tutorial are presented elsewhere (Volk et al., 2019). Briefly, the participants sat in relaxed upright position in front of a computer screen and followed a self-explanatory video tutorial. A human instructor explained and showed the following eleven facial expressions: Face at rest (no movement), wrinkling of the forehead, closing the eyes normally (gentle eye closure), closing the eyes forcefully (forceful eye closure), nose wrinkling, smiling with closed mouth, smiling with open mouth, lip puckering (pursing lips), blowing-out the cheeks (cheek blowing), snarling, and depressing lower lip. The participants performed each expression three times (three trials) before the next expression was explained. One session contained all expressions. On day t1 two sessions were performed with a time lag of about 20 min. The row data of t1 were already presented in the previous publication (Mueller et al., 2022). The same two sessions with the same time lag were repeated on day t2 14 days later (Figure 1).

### Facial surface electromyography (sEMG) registration

The sEMG protocol was published recently (Mueller et al., 2022). Briefly, a multi-channel EMG system (gain: 100, frequency range 10–1,861 Hz; sampling rate 4,096/s; resolution: 5.96 nV/bit; DeMeTec, Langgöns, Germany) was used for the sEMG recordings using monopolarly connected reusable surface electrodes (Ag-AgCl discs, diameter of 4 mm, DESS052606, GVB-geliMED, Bad Segeberg Germany). Electromyograms were recorded from both sides of the face. To account for artifacts, signals were centered and bandpass filtered between 10 and 500 Hz. A 50 Hz notch filter was applied to account for interferences from the electrical circuit. Two electrode arrangements were applied simultaneously: The schemes developed by Fridlund and Cacioppo (1986) and by Kuramoto et al. (2019) were used (Figure 1). In the following, the two schemes are labeled as “Fridlund” and “Kuramoto”. In total, 58 electrodes were placed on the face (including one ground and two connected reference electrodes). For the Fridlund scheme, from the monopolarly measured electrodes bipolar channels were calculated by subtracting the signals from the respective electrode pairs. Data for the Kuramoto scheme were monopolarly analyzed. sEMG amplitudes were quantified as mean rms values during the steady state contraction phases of every facial expression and sEMG channel. Between the two sessions on day t1 and t2, electrodes were not removed, i.e., all electrodes remained in place for the two sessions in one day.

**FIGURE 2:**
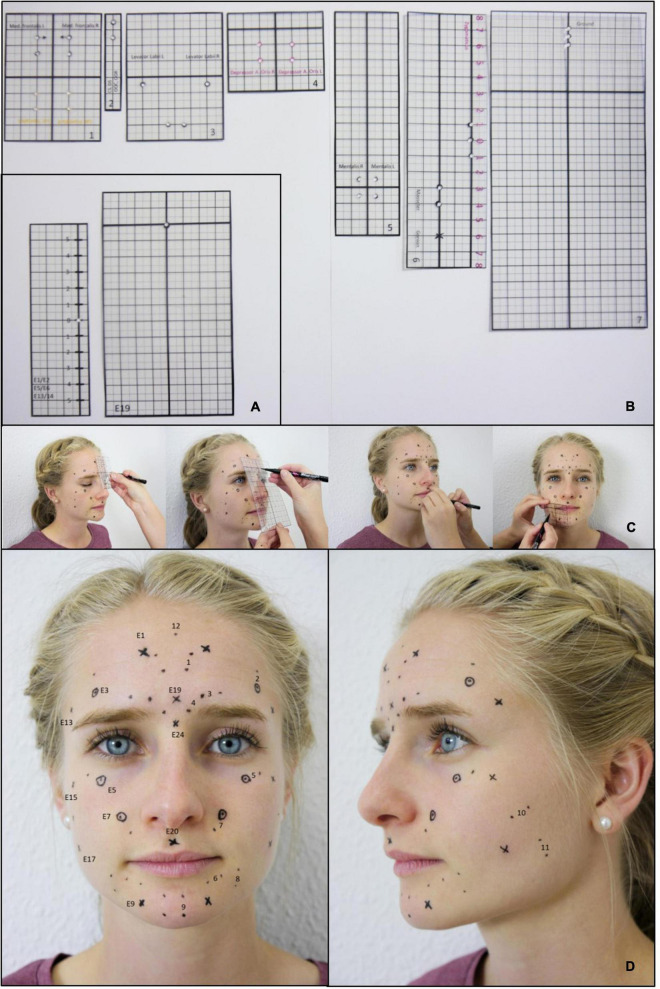
Standardized application of the EMG electrodes. **(A)** Templates for marking the electrode positions for the Kuramoto scheme. **(B)** Templates for marking the electrode positions for the Fridlund scheme. **(C)** Example for the process of marking the electrode positions. **(D)** Final result: Plot of all electrode positions for both, the Fridlund and the Kuramoto scheme. Labeled positions on the left side: Fridlund scheme (always positioned between the respective electrode pair). 1 = M. frontalis medial part, 2 = M. frontalis lateral part, 3 = M. corrugator supercilii, 4 = M. depressor supercilii, 5 = M. orbicularis oculi, 6 = M. orbicularis oris, 7 = M. levator labii superioris, 8 = M. depressor anguli oris, 9 = M. mentalis, 10 = M. zygomaticus major, 11 = M. masseter, 12 = Ground; right-sided positions: Kuramoto scheme: E1 = mid-frontal region, E3 = middle of eyebrow (= M frontalis lateralis like Fridlund), E5 = infrapalpebral sulcus (M. orbicularis oculi like Fridlund), E7 = nasolabial fold (M. levator labii superioris like Fridlund), E9 = M. mentalis, E13 = M. temporalis, E15 = cheek bone, E17 = hollow, E19 = glabella, E20 = philtrum, E24 = bridge of nose (left-sided positions are always labeled left + 1).

To ensure the use of identical electrode positions at t1 and t2, rigid laminated foils were used at both time points (Figure 2). Punched holes in the foils were used to mark the electrode positions on the face of the participants.

### Statistics

All statistical analyses were performed using IBM SPSS Statistics 25 (Chicago, IL, USA). Intraclass correlation coefficient (ICC) statistics expressed with lower and upper borders (i.e., minimal and maximal values) were used to analyze the retest reliability of the normalized EMG amplitudes between (a) the three trials in each session (intra-session reliability), (b) between the two sessions at one day (intra-day reliability), and (c) between the two days of measurement (between-day reliability). The higher the ICC value, the more precise is the estimate. ICC values less than 0.5 are indicative of poor reliability, values between 0.5 and 0.75 indicate moderate reliability, values between 0.75 and 0.9 indicate good reliability, and values greater than 0.90 indicate excellent reliability (Aniss and Sachdev, 1996). To allow a comparison to other data sets, the dimensionless coefficient of variation (CV) was calculated additionally (Koo and Li, 2016). The CV was calculated as the ratio of the standard deviation to the mean as a percentage ([CV = standard deviation/mean] × 100) for each individual and the different settings. The results are presented as means of the study sample. The lower the CV, the more precise was the estimate.

## Results

### Intraclass correlation coefficient statistics

In all tables minimum ICC values, the mean ICCs (values Fisher-z corrected, averaged, and the result inversely Fisher-z corrected), and the maximal ICC values are shown, respectively.

### Reliability of the sEMG recordings with the Fridlund scheme

Table 1 shows the results for the intra-session reliability, intra-day reliability, and the between-day reliability for all electrode positions. The mean intra-session ICCs were excellent for all electrode positions on both sides of the face (0.935–0.994). The least ICC values for the intra-session reliability occasionally were poor, but mainly moderate to good (0.117–0.891). The mean ICCs for the intra-day reliability were all moderate to good (0.674–0.881). The lowest ICC values for the intra-day reliability always showed poor values (0.011–0.488). The mean between-day ICCs values were poor to moderate (0.095–0.730). The minimum ICCs for the between-day reliability except one value always were poor (−0.152 to 0.298). Overall, systematic differences between the different muscles were not obvious for the Fridlund scheme. If poor values occurred they frequently could be detected on both sides.

**TABLE 1:** Intraclass correlation coefficient (ICC) statistics: Test and re-test reliability of the surface EMG recordings from the different facial muscles*.

	Intra-session reliability						Intra-day reliability						Between-day reliability							
	**Left**			**Right**			**Left**			**Right**			**Left**			**Right**				
	**Lower**	**Mean**	**Upper**	**Lower**	**Mean**	**Upper**	**Lower**	**Mean**	**Upper**	**Lower**	**Mean**	**Upper**	**Lower**	**Mean**	**Upper**	**Lower**	**Mean**	**Upper**		
**Fridlund**																				
DAO	0.867	0.971	0.993	0.891	0.978	0.998	0.200	0.864	0.978	0.249	0.881	0.980	0.556	0.730	0.880	0.040	0.650	0.927		
OOR	0.117	0.960	0.999	0.835	0.964	0.992	0.011	0.779	0.946	0.417	0.835	0.929	-0.009	0.591	0.901	0.069	0.668	0.854		
MENT	0.891	0.963	0.994	0.867	0.970	0.996	0.350	0.810	0.945	0.372	0.841	0.949	0.130	0.725	0.948	0.177	0.713	0.923		
MASS	0.695	0.965	1.000	0.306	0.952	1.000	0.138	0.710	0.982	0.060	0.674	0.961	0.072	0.561	0.961	0.137	0.550	0.951		
ZYGO	0.743	0.967	0.999	0.783	0.972	0.999	0.420	0.777	0.989	0.438	0.816	0.958	0.093	0.525	0.742	0.009	0.463	0.800		
LLSUP	0.778	0.965	0.994	0.728	0.961	0.992	0.438	0.874	0.955	0.422	0.843	0.956	0.164	0.625	0.879	0.298	0.689	0.874		
OOC	0.758	0.951	0.993	0.584	0.935	0.989	0.448	0.852	0.958	0.457	0.856	0.966	-0.140	0.605	0.942	0.171	0.649	0.910		
FROL	0.752	0.976	0.999	0.845	0.978	0.999	0.346	0.828	0.944	0.302	0.824	0.971	0.150	0.418	0.645	0.030	0.385	0.781		
FROM	0.833	0.982	0.997	0.846	0.981	0.999	0.447	0.857	0.991	0.488	0.849	0.992	0.117	0.545	0.812	0.118	0.433	0.893		
CORR	0.824	0.969	0.995	0.758	0.959	0.993	0.163	0.681	0.929	0.294	0.798	0.935	0.013	0.521	0.867	0.165	0.531	0.770		
DEPS	0.787	0.979	0.998	0.652	0.994	1.000	0.125	0.675	0.909	0.178	0.828	0.963	-0.152	0.351	0.840	-0.029	0.095	0.545		
**Kuramoto**																				
E9/E10	0.832	0.957	0.991	0.816	0.962	0.997	0.391	0.853	0.969	0.449	0.868	0.974	0.138	0.662	0.901	0.071	0.527	0.781		
E17/E18	0.831	0.966	0.997	0.801	0.966	0.996	0.553	0.868	0.973	0.651	0.867	0.958	0.353	0.679	0.916	0.291	0.643	0.924	**Color code**	
E15/E16	0.712	0.969	0.998	0.722	0.970	0.998	0.717	0.885	0.976	0.763	0.880	0.973	0.338	0.701	0.895	0.323	0.669	0.880	**lower border**	
E7/E8	0.865	0.964	0.997	0.863	0.969	0.998	0.464	0.883	0.959	0.458	0.881	0.965	0.117	0.702	0.912	0.169	0.689	0.899	poor	0.000
E5/E6	0.859	0.966	0.993	0.816	0.966	0.993	0.758	0.906	0.983	0.709	0.908	0.976	0.313	0.661	0.889	0.253	0.670	0.906	moderate	0.500
E13/E14	0.504	0.958	0.997	0.752	0.958	0.995	0.330	0.796	0.972	0.370	0.751	0.971	0.391	0.717	0.960	0.338	0.684	0.953	good	0.750
E1/E2	0.871	0.970	0.995	0.862	0.966	0.994	0.736	0.887	0.988	0.609	0.863	0.976	0.415	0.742	0.949	0.269	0.712	0.928	excellent	0.900
E3/E4	0.761	0.969	0.997	0.748	0.965	0.996	0.561	0.850	0.964	0.509	0.829	0.951	0.452	0.698	0.918	0.495	0.683	0.903	**upper border**	
**Central electrodes**																			poor	0.500
E20	0.801	0.966	0.996				0.651	0.867	0.958				0.291	0.643	0.924				moderate	0.750
E24	0.831	0.966	0.997				0.553	0.868	0.973				0.353	0.679	0.916				good	0.900
E19	0.831	0.966	0.997				0.553	0.868	0.973				0.353	0.679	0.916				excellent	1.000

*The higher the ICC value, the more precise the estimate.

DAO, depressor anguli oris; OOR, orbicularis oris; MENT, mentalis; MASS, masseter; ZYGO, zygomaticus; LLSUP, levator labii superioris; OOC, orbicularis oculi; FROL, frontalis lateralis; FROM, frontalis medialis; CORR, corrugator supercilii; DEPS, depressor superioris.

The color coding is explained on the right lower border of the table.

**FIGURE 3:**
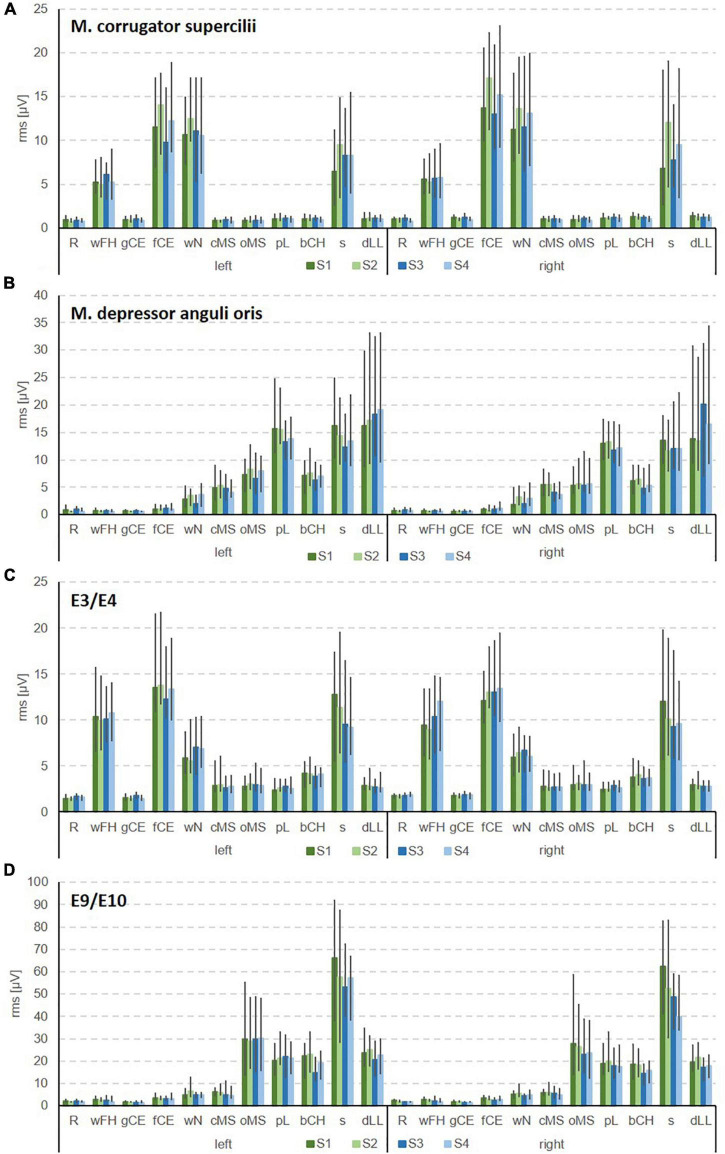
Examples for the bilateral sEMG for the Fridlund scheme **(A,B)** and the Kuramoto scheme **(C,D)**. **(A)** Recordings from M. corrugator supercilii. **(B)** M. depressor anguli oris. **(C)** E3/E4 in the frontal area. **(D)** E9/E10 in the chin area. R, face at rest (no movement); wFH, wrinkle forehead; gCE, closing the eyes normally (gentle eye closure); fCE, closing the eyes forcefully (forceful eye closure); wN, nose wrinkling; cMS, smiling with closed mouth; oMS, smiling with open mouth; pL, lip puckering (pursing lips); bCH, blowing-out the cheeks (cheek blowing), s, snarling; dLL, depressing lower lip; S1, session 1 at t1; S2, session 2 at t1; S3, session 1 at t2; S4, session 2 at t2.

Table 2 shows the results for all facial expressions. Two examples for the EMG recording results for all expressions are shown in Figures 3A, B. The mean intra-session ICCs were excellent for all facial expressions on both sides of the face (0.933–0.991). The lowest ICCs for the intra-session reliability rarely reached only poor values, but were mostly good to sometimes excellent (0.117–0.920). The mean ICCs for the intra-day reliability were all good to moderate (0.674–0.903). The lowest ICC values for the intra-day reliability mostly reached only poor results (0.011–0.577). The mean between-day ICCs values were poor to moderate (0.385–0.679). The lowest ICCs for the between-day reliability always were poor (−0.152 to 0.220). Overall, differences between the different exercises were not seen for the Fridlund scheme, but depressing lower lip somehow marked the lower border (compare with Table 2).

**TABLE 2A:** Intraclass correlation coefficient (ICC) statistics: Test and re-test reliability of the surface EMG recordings during the different facial movement tasks for the Fridlund and the Kuramoto scheme*.

	Intra-session reliability						Intra-day reliability						Between-day reliability							
	**Left**			**Right**			**Left**			**Right**			**Left**			**Right**				
	**Lower**	**Mean**	**Upper**	**Lower**	**Mean**	**Upper**	**Lower**	**Mean**	**Upper**	**Lower**	**Mean**	**Upper**	**Lower**	**Mean**	**Upper**	**Lower**	**Mean**	**Upper**		
**Fridlund**																				
Rest	0.778	0.988	1.000	0.584	0.989	1.000	0.337	0.674	0.851	0.399	0.712	0.951	-0.152	0.461	0.892	-0.025	0.401	0.897		
Wrinkle FH	0.783	0.964	0.991	0.839	0.971	0.998	0.377	0.757	0.916	0.427	0.785	0.878	-0.140	0.485	0.760	-0.029	0.499	0.857		
Gentle CE	0.858	0.989	0.999	0.891	0.991	1.000	0.138	0.693	0.955	0.303	0.750	0.957	-0.039	0.496	0.753	-0.022	0.415	0.837		
Forceful CE	0.695	0.949	0.984	0.612	0.950	0.982	0.200	0.798	0.958	0.060	0.756	0.946	0.130	0.664	0.879	0.118	0.553	0.846		
Wrinkle N	0.853	0.963	0.993	0.765	0.968	0.994	0.334	0.872	0.978	0.347	0.892	0.965	0.072	0.664	0.948	0.123	0.632	0.923		
Closed MS	0.863	0.964	0.998	0.803	0.972	1.000	0.187	0.805	0.966	0.178	0.834	0.980	-0.078	0.604	0.942	-0.022	0.583	0.902		
Open MS	0.920	0.980	0.998	0.884	0.983	1.000	0.125	0.850	0.947	0.509	0.903	0.971	0.117	0.618	0.927	-0.023	0.620	0.910		
Puckering L	0.743	0.957	0.993	0.306	0.956	1.000	0.277	0.844	0.991	0.453	0.862	0.987	0.055	0.532	0.812	-0.027	0.570	0.893		
Blow CH	0.747	0.958	0.993	0.773	0.959	1.000	0.048	0.835	0.989	0.438	0.875	0.963	-0.040	0.640	0.961	-0.025	0.679	0.951		
Snarl	0.869	0.963	0.998	0.919	0.965	0.994	0.060	0.870	0.958	0.577	0.874	0.992	0.009	0.657	0.894	0.220	0.604	0.836		
Depress LL	0.117	0.946	0.999	0.561	0.933	0.999	0.011	0.705	0.930	0.302	0.719	0.939	-0.021	0.385	0.796	-0.017	0.393	0.748		
**Kuramoto**																				
Rest	0.865	0.987	0.998	0.886	0.986	0.997	0.660	0.931	0.957	0.545	0.942	0.941	0.117	0.775	0.858	0.169	0.683	0.695	**Color code**	
Wrinkle FH	0.893	0.950	0.991	0.763	0.934	0.991	0.686	0.930	0.911	0.682	0.923	0.889	0.331	0.793	0.797	0.151	0.720	0.805	**lower border**	
Gentle CE	0.934	0.990	0.998	0.911	0.989	0.998	0.649	0.931	0.935	0.575	0.940	0.933	0.323	0.797	0.840	0.125	0.730	0.697	poor	0.000
Forceful CE	0.504	0.949	0.980	0.816	0.944	0.975	0.391	0.887	0.951	0.449	0.891	0.939	0.138	0.700	0.910	0.071	0.731	0.899	moderate	0.500
Wrinkle N	0.891	0.962	0.984	0.842	0.964	0.990	0.736	0.923	0.930	0.725	0.927	0.965	0.505	0.823	0.901	0.473	0.821	0.831	good	0.750
Closed MS	0.761	0.968	0.993	0.910	0.972	0.995	0.330	0.922	0.959	0.370	0.925	0.956	0.465	0.868	0.907	0.596	0.884	0.907	excellent	0.900
Open MS	0.889	0.976	0.993	0.863	0.977	0.996	0.744	0.968	0.983	0.672	0.973	0.976	0.575	0.851	0.912	0.594	0.849	0.866	**upper border**	
Puckering L	0.599	0.928	0.990	0.722	0.929	0.983	0.726	0.939	0.988	0.570	0.923	0.976	0.391	0.799	0.853	0.338	0.776	0.831	poor	0.500
Blow CH	0.865	0.952	0.986	0.899	0.950	0.990	0.724	0.953	0.976	0.750	0.945	0.973	0.652	0.939	0.960	0.607	0.925	0.953	moderate	0.750
Snarl	0.890	0.953	0.978	0.899	0.955	0.977	0.600	0.880	0.931	0.673	0.891	0.930	0.480	0.835	0.916	0.335	0.849	0.924	good	0.900
Depress LL	0.863	0.928	0.985	0.816	0.927	0.986	0.654	0.915	0.853	0.602	0.923	0.914	0.338	0.678	0.668	0.381	0.687	0.678	excellent	1.000

*The higher the ICC value, the more precise the estimate.

FH, forehead; CE, closed eyes; N, nose; MS, mouth smile; L, lips; CH, cheeks; open MS; LL, lower lips.

The color coding is explained on the right lower border of the table.

**TABLE 2B:** Intraclass correlation coefficient (ICC) statistics: Test and re-test reliability of the surface EMG recordings during the different facial movement tasks for the central electrodes*.

	Intra-session reliability			Intra-day reliability			Between-day reliability				
	**Lower**	**Mean**	**Upper**	**Lower**	**Mean**	**Upper**	**Lower**	**Mean**	**Upper**		
**Central electrodes**											
Rest	0.893	0.989	1.000	0.698	0.767	0.850	-0.050	0.235	0.622	**color code**	
Wrinkle FH	0.899	0.965	0.993	0.779	0.842	0.880	0.099	0.582	0.794	**lower border**	
Gentle CE	0.971	0.991	0.999	0.694	0.777	0.856	-0.003	0.362	0.621	poor	0.000
Forceful CE	0.700	0.937	0.966	0.417	0.762	0.881	0.495	0.749	0.826	moderate	0.500
Wrinkle N	0.911	0.965	0.981	0.763	0.891	0.922	0.579	0.722	0.877	good	0.750
Closed MS	0.922	0.977	0.995	0.784	0.839	0.946	0.299	0.725	0.848	excellent	0.900
Open MS	0.902	0.977	0.994	0.853	0.891	0.919	0.209	0.697	0.877	**upper border**	
Puckering L	0.875	0.968	0.993	0.858	0.918	0.960	0.053	0.637	0.816	poor	0.500
Blow CH	0.940	0.965	0.985	0.885	0.920	0.963	0.392	0.752	0.933	moderate	0.750
Snarl	0.844	0.946	0.975	0.171	0.776	0.880	0.291	0.739	0.867	good	0.900
Depress LL	0.862	0.935	0.991	0.764	0.821	0.856	0.049	0.533	0.772	excellent	1.000

*The higher the ICC value, the more precise the estimate.

FH, forehead; CE, closed eyes; N, nose; MS, mouth smile; L, lips; CH, cheeks; open MS; LL, lower lips.

The color coding is explained on the right side of the table.

### Reliability of the sEMG recordings with the Kuramoto scheme

Table 1 shows all results from the perspective of the electrode positions. The mean intra-session ICCs were excellent for all electrode positions when using the Kuramoto scheme (0.957–0.970). The lowest ICCs for the intra-session reliability reached moderate to good values (0.504–0.871). The mean ICCs for the intra-day reliability were all good (0.751–0.908). The lowest ICCs for the intra-day reliability reached poor to moderate and also two good values (0.291–0.763). The mean between-day ICCs values were all moderate (0.643–0.742). The lowest ICCs for the between-day reliability always were poor (0.071–0.495). Overall, clear differences between the different electrode positions were not seen for the Kuramoto scheme. The centrally positioned electrodes showed no other ICCs than the lateral ones.

The intra-session reliability of both schemes were equal. Compared to the Fridlund scheme, the mean ICCs and especially the lower border ICCs for the intra-day and between-day reliability were larger for the Kuramoto scheme.

Table 2 shows the results for all facial expressions when using the Kuramoto scheme. Two examples for the EMG recording results for all expressions are shown in Figures 3C, D. The mean intra-session ICCs were excellent for all facial expressions (0.927–0.991). The lowest ICCs for the intra-session reliability reached moderate to excellent values (0.504–0.971). The mean ICCs for the intra-day reliability were excellent to good (0.762–0.973). The lowest ICCs for the intra-day reliability reached poor to moderate values for the lateral electrodes (0.330–0.750), but the centrally positioned electrodes performed better with poor to good values (0.171–0.885). The mean between-day ICCs values were poor to good (0.235–0.868; exception for lateral electrodes at blowing cheeks: excellent). The lowest ICC values for the between-day reliability always reached poor to moderate values (−0.050 to 0.652). Overall, relevant differences between the different expressions were not seen for the Kuramoto scheme.

Again, the intra-session reliability of both schemes were equal. Compared to the Fridlund scheme, the mean ICCs and especially the lower border ICCs for the intra-day and between-day reliability were always better for the Kuramoto scheme when focusing on the facial expressions.

### Coefficient of variation statistics

The CVs are presented as means and standard deviation of the study group. The results are summarized in the Tables 3, 4. Overall, the results of the CV analyses confirmed the results of the ICC statistics.

**TABLE 3:** Coefficient of variation statistics: Test and re-test reliability of the surface EMG recordings from the different facial muscles*.

	Intra-session reliability				Intra-day reliability				Between-day reliability					
	**Left**		**Right**		**Left**		**Right**		**Left**		**Right**			
	**Mean**	**SD**	**Mean**	**SD**	**Mean**	**SD**	**Mean**	**SD**	**Mean**	**SD**	**Mean**	**SD**		
**Fridlund**														
DAO	9.61	6.59	9.31	9.02	20.83	14.49	19.80	13.48	25.99	19.75	24.75	23.50		
OOR	9.83	5.67	10.04	5.98	27.17	18.28	27.56	19.65	28.79	20.74	29.56	19.35		
MENT	13.56	9.76	11.55	7.34	36.18	21.89	36.05	20.96	26.24	17.59	31.62	19.57		
MASS	4.11	4.13	4.79	3.68	15.92	11.21	15.12	14.86	20.77	25.53	19.75	16.87		
ZYGO	4.84	3.61	4.65	3.40	14.05	10.91	15.85	10.18	23.03	17.62	23.43	20.69		
LLSUP	9.42	7.95	10.33	13.24	15.57	10.97	20.81	15.14	23.98	16.09	22.72	15.38		
OOC	9.54	8.58	14.09	8.68	18.58	11.86	14.10	11.40	26.91	19.22	19.08	12.91		
FROL	5.67	3.56	5.96	3.55	19.41	12.84	22.61	13.56	32.87	24.03	30.76	23.76		
FROM	3.64	3.12	3.32	4.22	13.99	9.85	16.06	10.73	29.13	23.70	27.90	22.21		
CORR	6.71	6.13	6.39	3.68	20.18	14.07	20.73	12.98	27.54	23.10	29.30	14.40		
DEPS	5.41	3.80	5.73	3.35	24.44	17.96	19.53	12.99	34.02	26.01	36.34	30.75		
**Kuramoto**														
E9/E10	8.68	6.26	7.42	4.95	19.13	15.18	15.86	12.70	19.96	14.70	23.46	15.82	**color code**	
E17/E18	7.22	4.40	5.41	3.24	10.57	4.77	9.96	5.64	16.66	16.08	21.00	15.80	worst	74.3
E15/E16	6.79	4.87	5.68	3.81	11.88	6.66	10.79	6.72	17.05	16.88	22.43	16.54		50.5
E7/E8	6.96	4.27	5.72	3.43	9.78	5.67	9.42	6.67	16.42	17.37	20.88	20.18		26.8
E5/E6	7.14	5.15	6.07	5.96	10.20	6.17	8.00	6.33	16.32	15.35	16.98	12.83	best	3.0
E13/E14	9.16	6.32	7.12	5.76	20.21	13.30	16.28	11.74	21.35	16.28	21.94	13.87		
E1/E2	7.15	4.54	5.29	3.76	15.26	12.53	9.81	8.02	19.29	16.62	15.92	13.32		
E3/E4	6.47	4.30	4.78	3.72	10.95	6.38	7.28	5.24	16.51	15.88	16.46	14.58		
**Central electrodes**														
E20	6.69	50,5	50,5		11.02	6.69			16.95	14.90				
E24	4.14	26,8	26,8		8.05	5.19			20.06	22.13				
E19	5.15	3,0	3,0		11.95	7.85			15.73	12.16				

*The lower the value of the coefficient of variation, the more precise the estimate.

DAO, depressor anguli oris; OOR, orbicularis oris; MENT, mentalis; MASS, masseter; ZYGO, zygomaticus; LLSUP, levator labii superioris; OOC, orbicularis oculi; FROL, frontalis lateralis; FROM, frontalis medialis; CORR, corrugator supercilii; DEPS, depressor superioris.

The color coding is explained on the right side of the table.

**TABLE 4A:** Coefficient of variation statistics: Test and re-test reliability of the surface EMG recordings during the different facial movement tasks for the Fridlund and the Kuramoto scheme*.

	Intra-session reliability				Intra-day reliability				Between-day reliability					
	**Left**		**Right**		**Left**		**Right**		**Left**		**Right**			
	**Mean**	**SD**	**Mean**	**SD**	**Mean**	**SD**	**Mean**	**SD**	**Mean**	**SD**	**Mean**	**SD**		
**Fridlund**														
Rest	7.49	6.73	7.83	7.42	20.57	15.65	20.75	15.60	56.04	37.44	26.84	20.90		
Wrinkle FH	12.97	9.77	12.76	9.77	19.81	15.63	19.53	14.47	39.44	22.59	27.73	20.79		
Gentle CE	7.09	6.50	6.71	5.92	20.01	15.40	18.26	13.05	58.06	34.80	25.33	20.28		
Forceful CE	16.45	13.41	16.30	12.77	20.16	18.07	420.39	6.95	30.98	29.38	28.79	20.08		
Wrinkle N	18.81	13.37	17.57	12.51	20.14	16.06	18.95	15.21	29.59	29.69	27.91	420.41		
Closed MS	16.61	12.50	16.43	12.39	20.45	16.34	18.66	15.79	74.33	40.76	28.96	22.13		
Open MS	13.86	10.80	13.45	10.69	16.21	13.94	16.29	13.60	53.56	34.78	27.60	21.40		
Puckering L	12.46	10.79	13.21	11.60	14.21	11.74	13.77	10.96	47.80	37.40	24.65	20.53		
Blow CH	17.04	12.12	18.47	13.13	17.26	13.48	17.18	12.78	46.65	30.93	25.94	20.57		
Snarl	21.03	13.14	20.79	12.29	420.46	16.11	19.97	16.40	45.71	31.11	29.52	22.02		
Depress LL	16.94	11.83	17.44	12.12	20.60	15.89	19.05	14.48	44.33	38.91	29.44	22.95		
**Kuramoto**														
Rest	7.45	5.09	5.94	4.47	13.50	10.29	10.93	8.79	17.94	16.07	19.88	15.58	**color code**	
Wrinkle FH	14.32	10.56	13.30	10.31	16.97	11.75	14.68	11.09	24.35	18.00	24.64	17.49	worst	74.3
Gentle CE	6.66	4.83	5.21	3.91	13.65	9.88	10.97	7.80	18.27	16.51	21.25	16.83		50.5
Forceful CE	14.70	12.59	13.81	11.58	13.78	11.58	3.15	10.81	19.51	13.86	18.82	13.93		26.8
Wrinkle N	14.28	9.32	13.00	8.83	14.52	9.94	13.34	9.67	21.73	14.30	20.86	14.42	best	3.0
Closed MS	15.07	8.52	14.07	8.97	15.52	15.50	13.99	13.19	22.60	15.71	20.41	14.23		
Open MS	13.18	9.09	12.40	9.14	13.13	11.20	13.14	11.12	20.98	14.87	22.14	15.47		
Puckering L	11.77	9.26	11.14	8.65	9.69	7.04	9.11	6.86	16.83	12.71	17.39	11.66		
Blow CH	16.89	8.19	16.29	8.45	12.52	9.72	12.97	9.58	19.39	13.27	19.24	12.73		
Snarl	18.16	10.71	17.61	10.42	16.27	13.77	16.29	13.60	20.42	15.22	20.37	14.92		
Depress LL	15.51	8.42	14.39	8.19	15.21	10.47	14.20	9.74	22.11	18.20	21.32	17.47		

*The lower the value of the coefficient of variation, the more precise the estimate.

FH, forehead; CE, closed eyes; N, nose; MS, mouth smile; L, lips; CH, cheeks; open MS; LL, lower lips.

The color coding is explained on the right side of the table.

**TABLE 4B:** Coefficient of variation statistics: Test and re-test reliability of the surface EMG recordings during the facial movement tasks for the central electrodes*.

	Intra-session reliability		Intra-day reliability		Between-day reliability			
	**Mean**	**SD**	**Mean**	**SD**	**Mean**	**SD**		
**Central electrodes**								
Rest	5.79	4.26	10.88	8.14	18.81	15.63	**color code**	
Wrinkle FH	13.08	9.83	14.29	10.40	23.74	17.10	worst	74.3
Gentle CE	5.22	3.60	11.52	7.99	19.27	16.43		50.5
Forceful CE	14.10	11.75	13.33	11.12	17.25	12.92		26.8
Wrinkle N	14.39	10.17	13.90	9.63	21.05	14.99	best	3.0
Closed MS	13.29	8.34	13.91	14.36	19.72	14.27		
Open MS	11.79	8.55	12.80	10.43	20.62	14.88		
Puckering L	11.09	8.95	8.89	6.65	16.52	13.03		
Blow CH	15.34	8.41	12.16	8.73	18.44	12.76		
Snarl	17.35	10.61	16.06	13.03	19.19	14.84		
Depress LL	14.98	9.47	14.31	9.98	20.73	16.87		

*The lower the value of the coefficient of variation, the more precise the estimate.

FH, forehead; CE, closed eyes; N, nose; MS, mouth smile; L, lips; CH, cheeks; open MS; LL, lower lips.

The color coding is explained on the right side of the table.

Regarding the recordings from different facial muscles, the CVs were good for the intra-session reliability for both schemes, but better for the Kuramoto scheme (range: minimal mean CV of 4.78 to maximal mean CV of 9.16 of all measurements) than for the Fridlund scheme (3.32–14.09). The intra-day reliability was mainly moderate for the Fridlund scheme (13.99–36.18), whereas the Kuramoto scheme (7.28–20.21) showed sometimes good estimates. The between-day reliability was moderate or below moderate for the Fridlund scheme (19.08–36.34), and moderate for the Kuramoto scheme (15.96–23.46). The central electrodes showed worse results for the intra-session reliability than the other electrodes (overall, 4.14–20.06).

Regarding the EMG recordings during the different facial movement tasks, the CV values were overall worse than for the recordings from different facial muscles. The CVs were good to moderate for the intra-session reliability for both schemes (Fridlund: 6.71–21.03; Kuramoto: 5.21–18.16). The intra-day reliability was moderate to good for both schemes, but better for the Kuramoto scheme (16.83–16.97) than for the Fridlund scheme (24.65–20.75). The between-day reliability was moderate or below moderate, or even worse for the Fridlund scheme (24.65–74.33), and moderate for the Kuramoto scheme (16.83–24.64). The central electrodes showed good to moderate results for all three interval settings (5.21–23.74).

Overall, the Kuramoto scheme produces better CV estimates than the Fridlund scheme.

## Discussion

Facial sEMG is an important and standard instrument for the evaluation of emotional expressions or as a diagnostic tool to analyze facial muscle function. Typically, participants or patients are examined several times, for instance by varying the stimuli, or at different days before and after an intervention (Hess et al., 2017). To detect differences in the EMG activity related to the experiment or clinical changes, it is important to guarantee a high test-retest reliability of facial sEMG, i.e., to rule out that different EMG findings at different recording instances are not just the result of the variability of the respective EMG recordings, as this would disqualify the method from clinical application.

Traditionally, the placement of the sEMG electrodes on the facial skin is oriented to topography of the subdermal topographical position of the facial muscles and the direction of the muscle fibers. The electrode scheme of Fridlund and Cacioppo (1986) is most frequently used. Pairs of electrodes are placed in a constant distance of 1 cm on 10 facial and the masseter muscle on each side of the face. The original publication contains detailed anatomical descriptions of the electrode positioning. While the distance between the electrodes of one pair is exactly defined by 1 cm, the anatomical description allows some variability. Therefore, it was very important to work with laminated foils with exactly defined electrode positions in the present study. At the same time this is a limitation, as it means that it might be necessary to use such foils in any psychophysical experiments with repeated measurements.

In general, it is surprising that test-retest reliability data for any sEMG scheme that is following the muscle topography (as the most frequent approach) are sparse. Demeco et al. (2021) analyzed the results of only four electrodes per side but wireless sEMG recording. Three trials of each participant were analyzed in one session with 4 min of rest between sessions. The exercises were: Frowning, closing eyes forcefully, showing teeth, and pursing lips. The test was repeated after 10 days. The intra-subject reliability (intra-session reliability was not analyzed) was very good for all the analyzed movements with 0.94 (CI 0.90, 0.98), i.e., lower than the intra-session reliability of the present study. Only Hess et al. (2017) used the test-retest methodology like in the present study. They measured a sEMG on the corrugator supercilii muscle (while frowning), orbicularis oculi m. (during wrinkles around the eyes), the levator labii superioris m. (lifting the upper lip in disgust), and the zygomaticus major (lifting the corners of the mouth while smiling) two times with a time interval of 15 or 24 months. Both studies cannot be directly compared because Hess et al. (2017) used images as stimuli material for non-voluntary reactions whereas we used video instructions for voluntary facial expressions. We assume that our instructions lead to more reproducible facial expressions. Emotional reactions should have a higher variability (Aniss and Sachdev, 1996; Rymarczyk et al., 2011). This might explain why the retest reliability was only high for the M. zygomatic major with ICC = 0.93, whereas the ICC were all <0.7 for all other settings in the study by Hess et al. (2017).

A critical factor during different sessions is the reproducible positioning of the electrodes when using the classical Fridlund scheme. Any repeated electrode application has also influences on inter-electrode distances and therefore electrode crosstalk variability. The EMG signal is highly sensitive to changes of the electrode positions with respect to the facial muscles (Frank et al., 2021). Therefore, it appears plausible that the test-retest reliability was better for the Kuramoto scheme. Recently, we have shown that the Kuramoto scheme performs better than the Fridlund scheme to differentiate distinct facial expressions (Mueller et al., 2022). This was surprising since any monopolar montage by nature contains more cross talk than a bipolar one (Mohr et al., 2018). The present study was the first to study the test-retest reliability of the Kuramoto scheme. Probably, especially this monopolar geometric and symmetrical electrode positioning is more robust against slight but unavoidable position changes. We can now conclude that the Kuramoto scheme is also the more suitable scheme when using facial sEMG for psychophysical experiments at repeated sessions with same participants. We did not perform a direct comparison of the Kuramoto scheme to high-density sEMG (HD sEMG) settings applying >90 electrodes with inter-electrode distance of ≤5 mm (Drost et al., 2006; Cui et al., 2020). At least the results for the intra-session reliability and intra-day reliability seem to be good enough to recommend the Kuramoto scheme for standard psychophysical experiments with small re-test intervals. HD sEMG is very time-consuming per experiment and probands. It remains open if HD sEMG can deliver a better re-test reliability, especially a better between-day reliability.

The present study has limitations. Using the video self-tutorial to demonstrate the facial movement tasks seems to us as the most reliable instruction technique (Volk et al., 2019). Nevertheless, it does not rule out a variable performance of the participants, as no feedback is implemented. It is proposed to evaluate only strikingly different but good defined facial expressions to minimize intra-individual variability (O’Dwyer et al., 1981; Demeco et al., 2021; Jung and Im, 2022). The disadvantage is that the probands perform artificial facial expressions when asked to perform the expressions demonstrated in the self-tutorial video. Furthermore, the preformed foils to mark the electrode positions, of course, still allow some remaining variability of the positioning of the electrodes. In the future, we plan to use screen-printed adhesive electrode arrays (Inzelberg et al., 2018). It looks like as these adhesive arrays allows a very reliable electrode placement, and therefore EMG recording (Gat et al., 2022). This seems important to us to establish an easy to use setting for psychophysical experiments especially when performed in large sample sizes or by non-EMG experts.

## Conclusion

High-resolution sEMG recordings of healthy probands showed an excellent intra-session test-retest reliability in regard of the Fridlund and the Kuramoto scheme, both in regard of the electrode positions and for the different facial expressions. The test-retest intra-day reliability and the between-day reliability was consistently better for the Kuramoto scheme. When using the Kuramoto scheme, a good to excellent mean intra-day and between-day reliability seems to be achievable.

## Data availability statement

The raw data supporting the conclusions of this article will be made available by the authors, without undue reservation.

## Ethics statement

The studies involving human participants were reviewed and approved by the Ethics Committee of the Jena University Hospital. The patients/participants provided their written informed consent to participate in this study. Written informed consent was obtained from the individual(s) for the publication of any potentially identifiable images or data included in this article.

## Author contributions

OG-L, GFV, and CA: conceptualization and supervision. OG-L and CA: first draft preparation. VT, NM, A-MK, and MH: data acquisition. VT, NM, and CA: data analysis. All authors contributed to the article and approved the final version.
